# Acute Intraprocedural Thrombosis After Flow Diverter Stent Implantation: Risk Factors and Relevance of Standard Observation Time for Early Detection and Management

**DOI:** 10.1007/s00062-022-01214-6

**Published:** 2022-09-06

**Authors:** Sophia Hohenstatt, Christian Ulfert, Christian Herweh, Silvia Schönenberger, Jan C. Purrucker, Martin Bendszus, Markus A. Möhlenbruch, Dominik F. Vollherbst

**Affiliations:** 1 Department of Neuroradiology, Heidelberg University Hospital, INF 400, 69120 Heidelberg, Germany; 2 Department of Neurology, Heidelberg University Hospital, INF 400, 69120 Heidelberg, Germany

**Keywords:** Cerebral aneurysms, Flow diversion, Intraprocedural thrombosis, Endovascular complications, Tirofiban

## Abstract

**Purpose:**

Acute intraprocedural thrombosis (AIT) is a severe complication of flow diverter stent (FDS) implantation for the treatment of intracranial aneurysms. Even though device-related thromboembolic complications are well known, there are no acknowledged risk factors nor defined surveillance protocols for their early detection. This study aimed to demonstrate that an angiographic active surveillance is effective to detect and treat AIT. Furthermore, we investigated risk factors for the occurrence of AIT.

**Methods:**

A prospective institutional protocol consisting of a defined observation period of 30 min following FDS deployment was established to detect AIT. Overall incidence, as well as the efficacy and safety of AIT treatment were assessed. Moreover, radiological and clinical outcomes of patients with AIT were analyzed. The influence of various patient- and procedure-related factors on the occurrence of AIT was investigated using multivariable forward logistic regression.

**Results:**

During active surveillance twelve cases of AIT were observed among a total of 161 procedures (incidence: 7.5%). The median time of first observation was 15.5 min (IQR 9.5) after FDS implantation. The early recognition of AIT ensured a prompt treatment with intravenous application of a glycoprotein IIb/IIIa inhibitor, which led to complete thrombus resolution in all cases without hemorrhagic complications. Patients with pre-existing arterial hypertension and side branches originating from the aneurysmal sac had a higher risk of AIT (respectively OR, 9.844; OR, 3.553). There were two cases of re-thrombosis in the short-term postoperative period, of whom one died. The remaining patients with AIT had a good clinical outcome.

**Conclusion:**

Active surveillance for 30 min after FDS implantation is an effective strategy for early detection and ensuing treatment of AIT and can thus prevent secondary sequalae. Hypertension and side branches originating from the aneurysmal sac may increase the risk of AIT.

**Supplementary Information:**

The online version of this article (10.1007/s00062-022-01214-6) contains supplementary material, which is available to authorized users.

## Introduction

Despite increasing clinical experience and technological progress, procedural complications still occur occasionally in the endovascular treatment of intracranial aneurysms with thromboembolic complications representing the leading cause of morbidity and mortality [1].

Flow diversion has revolutionized the treatment of a large number of intracranial aneurysms in the past years, extending the indications to aneurysms that were previously difficult to treat with traditional endovascular techniques [2]. However, especially flow diverter stents (FDS) carry a relatively high risk of thromboembolic complications since they have a much higher metal mesh density compared to other conventional stents. This increases the risk of thrombus formation within the stent, potentially leading to stent occlusion, distal embolism, and occlusion of vessels originating from the aneurysm or the parent vessel which is covered by the stent [3].

Prevention of clot formation is the most effective way to avoid thromboembolic complications; however, once a thrombus forms immediate action is require as an untreated clot can enlarge and lead to irreversible ischemic complications downstream. Early angiographic signs, such as a contrast filling defect within or adjacent to the stent or the occlusion of covered side branches, allow the detection of acute intraprocedural thrombosis (AIT) after insertion of the FDS [4]. Prompt pharmacological treatment with immediate intravenous administration of a glycoprotein IIb/IIIa receptor inhibitor can effectively prevent further thrombus formation and subsequent ischemic brain damage by promptly dissolving the thrombus [5, 6]. Therefore, an active monitoring protocol after implantation of FDS was established in our department to identify any spontaneous AIT during the procedure and treat it accordingly. This study aimed to demonstrate that an angiographic active surveillance time is justified and effective to detect and treat AIT. Furthermore, we analyzed several patient-related and procedure-related factors to investigate risk factors for the occurrence of AIT.

## Material and Methods

### Patient Selection

An institutional protocol consisting of an observation period of 30 min following FDS was established in 2014. Out of our prospectively maintained institutional neurointerventional database we retrospectively identified all consecutive adult patients who underwent FDS implantation for the purpose of aneurysm treatment between 2014 and 2021. Out of these patients we further selected and analyzed those who developed AIT. AIT was defined as angiographic evidence of occlusive or non-occlusive thrombus formation within the FDS or occlusion of covered side branches seen on the control angiography that is routinely performed immediately and in 5–10 min intervals after FDS deployment to confirm correct vessel wall apposition and rule out AIT or other complications.

### Data Collection

Data collection and analysis was approved by the institutional Ethics Committee. We collected data on patient demographics (including age and sex), clinical data (including clinical presentation, complications, and outcome), aneurysm characteristics (including size, location and morphology), as well as FDS specifications (including type and size), antiplatelet regimen and dosing of intravenous (i.v.) tirofiban (a GpIIb/IIIa antagonist), if applied.

Aneurysmal occlusion rates were assessed using the O’Kelly Marotta scale (OKM) or the Raymond-Roy occlusion classification (RROC) for invasive catheter angiography and magnetic resonance imaging (MRI) scans, respectively. The clinical evaluation was performed by a certified neurologist using the modified Ranking Scale (mRS) before treatment, at discharge and during follow-up. A score of 0 and 1 was assumed as a good functional outcome.

We included aneurysm treatment in the anterior and posterior circulation, as well as ruptured and unruptured aneurysms.

### Procedure

All procedures were performed with the patient under general anesthesia and patients were transferred to the neurocritical care ward after treatment. Patients who received elective treatment received standardized dual antiplatelet therapy (DAPT) with aspirin (ASA; 100 mg daily) and clopidogrel (75 mg daily) for at least 5 days prior to the procedure. Platelet reactivity testing was routinely done before the elective procedures to adopt the best pharmacological treatment in cases of reduced efficacy of clopidogrel. In these instances, patients were loaded either with ticagrelor or prasugrel. During the procedure, patients were on anticoagulation using heparin to achieve an activated clotting time > 250 s. Patients treated in an emergency setting received periprocedural and postprocedural treatment with tirofiban (dosage adapted to the patient’s weight according to the instruction for use). If a patient developed AIT i.v. tirofiban was administered as soon as possible. The maintenance dosage was given for 6–24 h, followed by the intended oral antiplatelet drugs. Before groin closure all patients received non-enhanced flat-panel computed tomography (CT) scans to rule out hemorrhagic or other complications. All patients received DAPT, at least for the first 3 months following the procedure. Afterwards single antiplatelet therapy was continued. All patients received follow-up clinical assessments and MRI and/or cerebral angiography at least after 3–6 months and 12 months.

### Statistics

SPSS Statistics, Version 25.0 (IBM, Armonk, NY, USA) was used for statistical analysis. Quantitative data are presented as number (relative frequency) or mean ± standard deviation (SD). To investigate the influence of patient-related and procedure-related factors on the occurrence of AIT, we analyzed all selected variables using binary logistic regression models, with acute in-stent thrombosis as the outcome variable. Subsequently, only the significant variables (*P* < 0.05) of this analysis were used in an interim multivariable logistic regression model. This model was adjusted with a variable selection based on the *P*-value with a forward stepwise approach based on the Wald test, resulting in the final multivariable logistic regression model. Two-sided *P*-values of 0.05 were defined as the threshold for statistical significance and were not adjusted for multiple testing due to the hypothesis-generating fashion of the study. Hence, the *P*-values should be interpreted descriptively. For odds ratios (OR), 95% confidence intervals (95% CI) were calculated.

## Results

### Patient Cohort

A total of 152 consecutive patients (74% female; mean age 56 ± 11.7 years) with 187 aneurysms who were treated in 161 procedures with FDS at our center between 2014 and 2021 matched the inclusion criteria of this study. Patient and aneurysm characteristics are summarized in Table 1.

**Table 1:** Main characteristics of the studied population including patient, aneurysms and procedure-related characteristics as well as the main outcomes of the radiologic and clinical follow-up

**Patient characteristics**									
*No. of patients*	152								
*Patient age*	56 ± 12 years (22–81 years)								
*Gender*	Female 112 (74%)					Male 39 (26%)			
*Comorbidities*	Arterial hypertension 88 (58%)			DM II 13 (9%)		Hypercholesterolemia 66 (43%)		Declared smokers 59 (38.8%)	
**Aneurysm characteristics**									
*No. of aneurysms*	187								
*Aneurysm location*	Extradural ICA 21 (11.2%)		Intradural ICA 119 (63.6%)		ACA and ACOM 17 (9.1%)	MCA 8 (4.3%)	Vertebrobasilar 22 (11.8%)		
*Morphology*	Saccular 156 (83.4%)			Fusiform 19 (10.2%)		Dissecting 4 (2.1%)		Blister 8 (4.3%)	
*SAH*	Non-ruptured 161 (86.1%)			Acute 6 (3.2%)		Subacute (< 2 weeks) 2 (1.1%)		Old (> 2 weeks) 14 (7.5%)	
*Aneurysm size* *(maximum diameter)*	8.9 ± 7.6 mm (1–60 mm)								
*Diameter of the parent artery*	Proximal 3.4 ± 0.9 mm (1–5.5 mm)					Distal 3.0 ± 0.8 (1–5.5)			
**Procedural details**									
*No. of procedures*	161								
*No. of implanted FDS*	170								
*Type of FDS*	FRED 86 (50.6%)	FRED X 28 (16.5%)	FRED Jr. 14 (8.2%)	PED 11 (6.5%)	PED Shield 4 (2.4%)	PED Vantage 21 (12.4%)	P48HPC 2 (1.2%)	SILK 1 (0.6%)	SURPASS 3 (1.8%)
*Technique*	FDS only 113 (70.2%)			FDS + coiling 45 (27.9%)			FDS + other 3 (1.9%)		
*In-stent PTA*	Yes 8 (5.0%)					No 153 (95%)			
*Mean procedural time*	106 ± 50 min (40–269 min)								
**Complications**									
*Total*	28 (17.4%)								
	Transient ND 19 (11.8%)					Persistent ND at discharge 3 (1.9%)			
*Thromboembolic*	24 (14.9%)								
	Intraoperative 12					Postoperative 12			
*Hemorrhagic*	4 (2.5%)								
**Radiologic and clinical follow-up**									
*Occlusion rates at latest follow-up (RROC)*^*6*^	I: Complete occlusion 124 (66.3%)		II: Residual neck 28 (14.97%)		III: Residual aneurysm 16 (8.56%)		No follow-up available 19 (10.16%)		
*Clinical outcome at latest clinical follow-up (mRS)*	Good (mRS 0–1) 148 (97.4%)			Poor (mRS ≥ 2) 2 (1.3%)			Death (mRS 6) 2 (1.3%)		

Data indicated as mean ± standard deviation (minimum—maximum) or absolute number of cases (relative frequency in %). Relative frequency related to the number of patients (*n* = 152) or to the number of aneurysms (*n* = 187) for the “patients” or “aneurysm” sections, respectively

*no.* number, *DM II* diabetes mellitus type 2, *ICA* internal carotid artery, *ACA* anterior cerebral artery, *ACOM* anterior communicating artery, *MCA* middle cerebral artery, *SAH* subarachnoid hemorrhage, *FDS* flow diverter stent, *PTA* percutaneous transluminal angioplasty, *ND* neurological deficit, *RROC* Raymond-Roy occlusion classification, *mRS* modified Ranking Scale

Most aneurysms (70%) were treated with flow diversion only, while 28% received adjunctive coiling. The rationale for adjunctive coiling was a maximum size > 10 mm, an irregular shape or an acute aneurysm rupture. In 3 patients a combination with other devices was used: 2 woven endobridge (WEB) devices (MicroVention, Aliso Viejo, CA, USA) and 1 Solitaire AB Stent (Medtronic Neurovascular, Irvine, CA, USA). In-stent percutaneous transluminal angioplasty (PTA) to open the FDS completely was necessary in 8 cases. The main specifications of the used FDS as well as procedural details are summarized in Table 1.

Out of 187 aneurysms, 141 (75.4%) had a follow-up at 1 year with a complete or near-complete occlusion rate of 90.1% (OKM1 68.1% and OKM2 22%). At the last clinical follow-up, 97.4% of patients had a good clinical outcome defined as mRS 0‑1.

### Acute Intraprocedural Thrombotic Complications

**Fig. 1:**
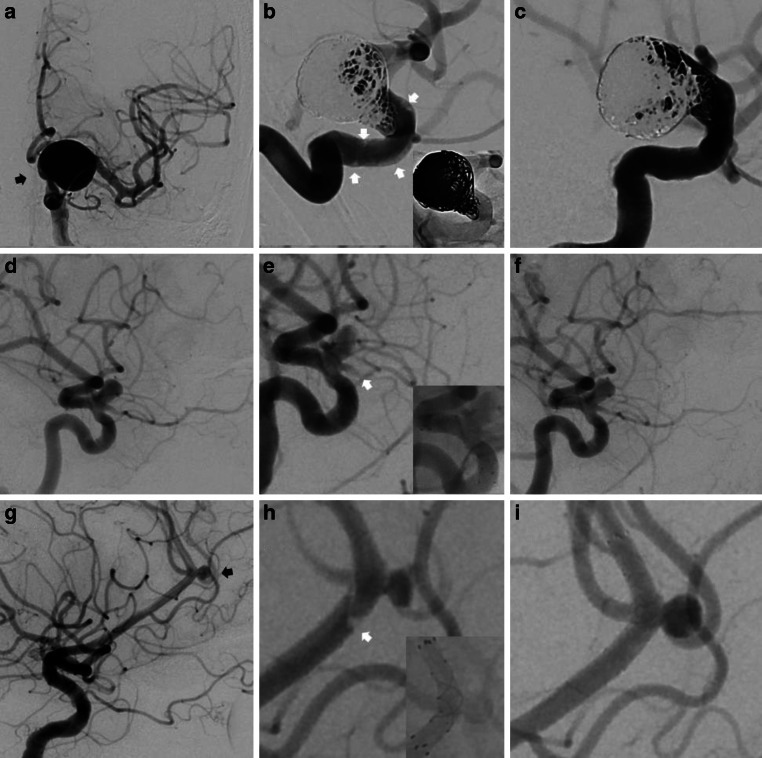
Three illustrative cases*. ***a** Subtracted AP view of an unruptured aneurysm of the supraophthalmic internal carotid artery (*black arrow*). **b** Subtracted and unsubtracted magnified view after coiling and Pipeline Vantage implantation with evidence of in-stent apposition thrombi (*white arrows*). **c** Subtracted magnified view after i.v. tirofiban demonstrating complete thrombus dissolvement within the stent. **d** Subtracted lateral view of a supraophthalmic aneurysm. **e** Subtracted and unsubtracted magnified view after FRED implantation. Thrombosis of the covered ophthalmic artery (*white arrows*). **f** Subtracted magnified view after i.v. tirofiban demonstrating complete thrombus dissolvement. **g** Subtracted lateral view of an unruptured saccular pericallosal aneurysm (*black arrow*). **h** Subtracted and unsubtracted magnified view after FRED Jr. implantation with partially occlusive thrombus visualized at the proximal end in correspondence to the transition zone of the flared ends with the flow diverting segment of the stent (*white arrow*). **i** Subtracted magnified view after i.v. tirofiban demonstrates complete thrombus dissolvement

**Fig. 2:**
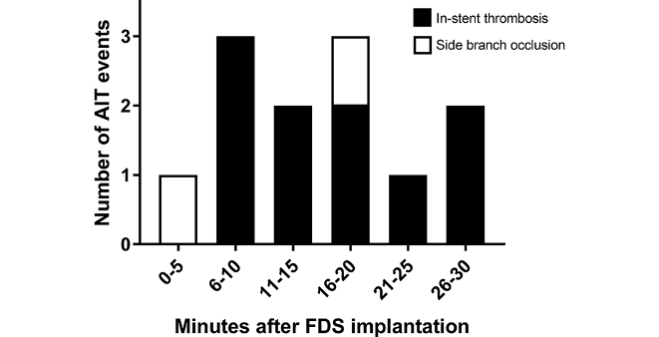
Time-event chart of the chronology of AIT detection for each patient. The X‑axis indicates the timing in five mite intervals in which a control angiogram was performed to detect AIT. The Y‑axis pinpoints the number of observed AIT events distinguishing between in-stent thrombosis (*black*) and side branch occlusion (*white*). Acute intraprocedural thrombosis (*AIT*); flow diverter stent (*FDS*)

A total of 12 cases of AIT in 12 patients (83% female; mean age 59 ± 10.9 years) with 13 aneurysms were identified among the total of 161 procedures included in this study (incidence: 7.5%). The case summaries for these procedures are presented in Table 2. Three illustrative cases are shown in Fig. 1. In 10 patients contrast filling defects in digital subtraction angiography (DSA) were observed within the stent, corresponding to an in-stent thrombosis. In the remaining 2 cases, side branch occlusions covered by the FDS were detected. Median interval of thrombosis after FDS deployment was 15.5 min (IQR 9.5min). Fig. 2 illustrates in graphic form the chronology of AIT detection for each patient. All patients were promptly treated with a bolus of i.v. tirofiban (dosage adjusted to body weight), which rapidly led to complete angiographic resolution of AIT in all cases. Tirofiban was then continued for 6–24 h (maintenance dose). In the case of the acutely ruptured aneurysm (patient no. 7) apposition thrombi formed despite the i.v. tirofiban bolus given before the FDS deployment. After an angiographic observation time of 30 min they resolved spontaneously without the need of adjunctive pharmacological measures.

**Table 2:** Case summaries of patient cohort with acute thrombotic complications—patient, aneurysm and treatment characteristics

Patient characteristics			Aneurysm characteristics					Treatment characteristics			
Patient	Age^a^ [years]	Risk factors	SAH	Location	Morphology	Max. diameter [mm]	Parent artery mean diameter [mm]	FDS	Periinterventional antiplatelet therapy	Technique	Time between FDS deployment and thrombosis [min]
1	60	aHT	No	Intradural ICA	Saccular	24.9	3.1	FRED	ASA + Clopi	FDS + coiling	14
2	50	aHT, HCho, smoke	No	ACA	Saccular	3.8	2.0	FRED Jr	ASA + Clopi	FDS	30
3	70	aHT, HCho, smoke	No	Intradural ICA	Saccular	6.9	2.6	FRED	ASA + Clopi	FDS	17
4	40	aHT, HCho, smoke	No	ACOM	Saccular	5.6	2.2	FRED Jr	ASA + Clopi	FDS	5
5 (2 aneurysms)	60	aHT	No	Extradural ICA (both)	Saccular (both)	4.5; 2.5	4.0	FRED	ASA + Tica	FDS	10
6	50	aHT, smoke	No	Vertebrobasilar	Dissecting	5.5	2.2	FRED Jr	ASA + Clopi	FDS	30
7	60	aHT, HCho, smoke	Yes, > 2 weeks	Intradural ICA	Saccular	3.5	3.6	FRED	Tirofiban	FDS	10
8	70	aHT, HCho, smoke	No	Extradural ICA	Saccular	15.5	3.8	FRED	ASA + Clopi	FDS + coiling	11
9	50	aHT, HCho, smoke	No	MCA	Saccular	11.0	2.9	FRED X	ASA + Pra	FDS	18
10	70	aHT	No	Intradural ICA	Saccular	7.1	3.9	FRED X	ASA + Clopi	FDS + coiling	24
11	70	aHT, HCho, smoke	No	Intradural ICA	Saccular	4.7	3.9	FRED X	ASA + Clopi	FDS + coiling	18
12	70	aHT, HCho	No	Intradural ICA	Fusiform	19.7	3.8	PED Vantage Shield	ASA + Clopi	FDS + coiling	6

*F* female, *M* male, *aHT* arterial hypertension, *HCho* hypercholesterolemia, *SAH* subarachnoid hemorrhage, *ICA* internal carotid artery, *ACA* anterior carotid artery, *ACOM* anterior communicating artery, *MCA* middle cerebral artery, *no.* number, *FDS* flow diverter stent, *ASA* aspirin, *Clopi* clopidogrel, *Tica* ticagrelor, *Pra* prasugrel

^a^ Exact age is not reported for anonymization purpose.

Most patients (83%) had a favorable clinical outcome, except for two. One patient (patient no. 12) died due to a large ischemic stroke, caused by a complete rethrombosis of the FDS 5 days after the treatment of an unruptured fusiform aneurysm of the internal carotid artery. Lacking compliance with the antithrombotic medication was suspected as the most likely cause for the rethrombosis in this patient. Another patient (patient no. 3) had an mRS 4 at long-term follow-up because of progression and rupture of another aneurysm besides the treated aneurysm 3 years after the implicated procedure.

Another case of rethrombosis of a FDS occurred in patient no. 11 1 week after discharge. In this patient, an aneurysm of the posterior communicating artery had been treated with FDS implantation in the ICA and adjunctive coiling. Thrombosis was first suspected on MRI due to mild headache and was immediately confirmed by a flat-panel CT and DSA 8 days after FDS implantation. The patient promptly received i.v. tirofiban and low-molecular heparin, followed by an antiplatelet therapy with daily ASA and prasugrel, as well as rivaroxaban (the prior DAPT consisted of ASA and ticagrelor). The patient recovered with no sequelae and had an mRS 0 at the 1‑year follow-up along with a patent stent and complete occlusion of the aneurysm (OKM 1).

There were no hemorrhagic complications related to treatment of AIT in this patient cohort.

All patients who developed AIT, except for the one with the acutely ruptured aneurysm (patient no. 7), received preprocedure platelet reactivity testing leading to nine patients receiving DAPT consisting of ASA in combination with clopidogrel. The remaining two patients who were non-responders to clopidogrel received ASA in combination with ticagrelor and ASA in combination with prasugrel, respectively (Table 2).

Long-term follow-up (≥ 1 year) was available for all patients with AIT, except for patient no. 12. At the latest imaging follow-up 9 patients (75%) had a complete occlusion of their aneurysm (OKM 1), patient no. 2 had a neck remnant (OKM 2) and patient no. 9 had a residual perfusion of the aneurysm (OKM 3).

The results of the analysis of the influence of several patient-related and procedure-related factors on the occurrence of AIT including the results of the multivariable logistic regression model are summarized in the Supplemental Table 1 and Supplemental Table 2. Two of the investigated parameters showed a significant positive correlation with AIT occurrence in the multivariable model: arterial hypertension (aHT; OR, 9.844; 95% CI, 0.013–0.808; *P* = 0.031) and the presence of a side branch originating from the aneurysm (OR, 3.553; 95% CI, 0.082–0.963; *P* = 0.043). For aneurysms with a wide neck configuration (> 4 mm) the rate of AIT tended to be higher, however, without reaching statistical significance (*P* = 0.124).

Of the overall FDS population the incidence of AIT in patients suffering from pre-existing aHT (*n* = 88) amounts to 13% versus only 2% in the cohort with a physiological blood pressure (*n* = 64). When considering all the patients with an AIT 11 out of 12 (91.7%) had a history of aHT. Furthermore, acute thrombosis occurred in 15.2% versus 5.2% in aneurysms with (*n* = 33) and without (*n* = 154) a side branch originating directly from the aneurysm, respectively. In the AIT subgroup, 5 out of 13 aneurysms (38.5%) had a side branch originating directly from the aneurysm.

## Discussion

In this study, using a systematic angiographic active surveillance protocol after FDS implantation, we could demonstrate that AIT is present in 7.5% in the early stage after FDS implantation. This complication can be early detected, and safely and effectively be treated to prevent ischemic complications. Furthermore, hypertension and side branches originating from the aneurysmal sac were identified as potential risk factors for the occurrence of AIT.

Ischemic stroke due to thromboembolism is the most frequent perioperative and postoperative complication associated with FDS implantation [7]. The reported stroke rates vary between 4% and 6% [8–10]. Thromboembolic complications can be divided in acute intraprocedural, subacute postprocedural and delayed complications [7]. In the case of AIT, immediate action is required, as an untreated clot can enlarge and quickly progress to occlude both the FDS and the parent vessel, and subsequently lead to a disabling stroke due to ischemia of the downstream vascular territory.

Despite all reasonable precautions being taken, such as appropriate pharmacological preparation and platelet reactivity testing, a subgroup of patients still develop acute thrombosis. Therefore, early recognition and subsequent treatment of AIT is a major factor for good outcome. There are currently no recommendations regarding a regular waiting time after FDS implantation and many centers terminate the procedure after an inconspicuous DSA instantly after FDS placement. The average time, after which AIT was detected in our study, was 16 min, which shows that AIT does by far not always occur immediately. Indeed, we experienced two cases of AIT at the 30 min control angiogram which raises the consideration that even a longer observation time could be justified. If the intervention is ended prematurely, the risk of missing such a relevant complication, that can be prevented by active surveillance followed by immediate treatment, is high. Since most patients are treated while under general anesthesia, most neurological deficits are detected not until the patient is awake again, which may imply a structural, often not fully reversible damage to the brain parenchyma that could potentially have been avoided. We strongly believe that these arguments are sufficient to justify the prolongation of the procedure for 30 min after device deployment for active angiographic surveillance after FDS implantation.

We observed two cases of later stent rethrombosis in this series with an incidence of 16.7% (related to the 12 patients who developed AIT). Therefore, we suggest additional careful clinical surveillance in the first postoperative days, as well as instructing the patient to promptly seek medical attention in case of new neurological symptoms.

Interindividual variable response to antiplatelet therapy [11], unpredictable patient compliance with medication, potential drug interactions, and the presence of comorbidities are already known to be linked to a higher risk of thromboembolic complications, but there are most likely other multifactorial variables [12]. Risk factors for AIT, specifically after FDS placement, have been scarcely investigated so far and the data we rely on come largely from the cardiologic field. A study investigating predictors of ischemic stroke after intraprocedural thrombosis found that current smoking was the only independent predictor [13]; however, to draw a conclusion not only based on flow diversion (pipeline embolization device) but also on stent-assisted coiling. Cardiologists report conflicting data regarding a history of aHT, for which both positive and negative associations with device-associated thromboses have been reported. In our multivariate regression analysis, we found a higher rate of AIT in patients with known aHT and in the presence of a side branch originating from the aneurysm. In one study, aHT was shown to be a protective factor for thrombosis of cardiologic drug-eluting stent (DES); however, only for delayed and not early occurrence [14]. On the other hand, different studies unanimously reported that high scores in the CHA2DS2-VASc score, which incorporates important cardiovascular risk factors as aHT, correlates with a higher incidence of DES thrombosis [15, 16]. Regarding the side branches originating from the aneurysm, further investigations are needed to better understand the effects of physical and mechanical properties of the stent on the dynamics of blood flow in this particular angioarchitecture. Interestingly, there was no increased rate of AIT in small parent vessels (mean diameter < 2.5 mm). The fear of a higher risk of stent occlusion, acute and delayed, given the lower volume flow rate and higher metal-to-cross-sectional flow area ratio, represents to date one of the main concerns to use FDS in the distal vasculature [17]. Another interesting aspect is that no cases of AIT were seen in the acute SAH patients, where there is a known hypercoagulable state [18].

The reported complication rate for AIT after FDS placement varies between 2% and 6% with most data relying on the PED (Medtronic Neurovascular) [4, 6, 9, 19, 20], which is the most widely used FDS in the USA. A recently published multicenter analysis about the FRED flow diverter (MicroVention) reported a similar complication rate of 5.5% [21]. The rate in our study was slightly higher at 7.5%; however, we considered not only thrombosis exclusively limited to the stent lumen but also acute occlusion of covered side branches, which are also caused by thrombus within or directly adjacent to the stent. The latter is reported to occur in about 2% of patients treated with PED [22] and up to 5% when considering different FDS [23]. Prevention of device-associated thrombosis is one of the strongest drivers of innovation, and lately new FDS with allegedly less thrombogenic materials or antithrombotic coatings are being developed, showing promising preliminary results [24]; however, further systematic studies are still lacking so far.

Various rescue strategies are available with i.v. antiplatelet drugs representing the first-line approach due to the platelet-rich composition of the acutely formed thrombi [6, 7]. Different glycoprotein (GP) IIb/IIIa receptor antagonist are available for this purpose: abciximab (ReoPro, Eli Lilly Inc.), tirofiban (Aggrastat, Medicure International Inc.), and eptifibatide (Integrilin, Millenium Pharmaceuticals Inc.). These drugs have different pharmacokinetic and pharmacodynamic properties, nevertheless, they seem to have comparable efficacy and safety profiles even if direct comparison studies are lacking. Usually, these drugs are given intravenously, although some authors report likewise efficacy of intra-arterial administration with the advantage of a lower overall dosage [4, 6, 7]. Intracranial hemorrhage is one of the main associated risks of antiplatelet medications. Even though we had none of these in our series it is reported to be as high as 7% (2/30 cases) [6], thus all glycoprotein (GP) IIb/IIIa receptor antagonists should be applied with great caution. Further on, if thrombus dissolvement cannot be achieved pharmacologically, other actions, such as direct thrombus aspiration or stent retriever thrombectomy through the stent lumen might be required, which was not necessary in this study.

An arguable drawback of an active surveillance strategy is the augmentation of the radiation dose. The whole treatment usually requires only a few DSA runs in total in an uncomplicated FDS implantation. Adding several DSA runs for active surveillance during the 30 min control phase, can significantly increase the radiation dose. Therefore, to answer the question if the benefit of early AIT detection outweighs this increase in radiation dose, further studies are needed. After further investigation of risk factors for AIT, in the future, the presented active surveillance protocol could be tailored dependent on the individual patient risk for AIT.

The main limitation of this report is the institution-based, nonrandomized, retrospective cohort design of the study. Furthermore, the angiographic and clinical data were not analyzed by an independent core laboratory and no control group was used for comparison. Another important limitation is that two types of FDS were predominately used (FRED and PED), even though many other FDS are available on the market; however, we still assume that our results may be representative, as precisely these FDS types are currently the most used worldwide. Additionally, to our knowledge, this is the only study presenting a systematic active surveillance protocol after FDS placement to date.

## Conclusion

Active surveillance for 30 min after FDS implantation is an effective strategy for the early detection and the subsequent treatment of AIT, which was present in about 7% of patients. Early identification and treatment of this complication by systemic application of Gp IIb/IIIa antagonists is highly effective, preventing subsequent disabling sequelae. Hypertension and side branches originating from the aneurysmal sac were independent risk factors for AIT; however, further studies are needed to confirm and better understand the underlying pathophysiological mechanisms.

## Supplementary Information

The supplementary material includes a detailed table about the influence of various patient-, aneurysm- and treatment-related variables on the acute intraprocedural thrombus formation. The summary of the univariate models as well as the final multivariable logistic regression model is included.
