# Supplementary material for: Acute Intraprocedural Thrombosis After Flow Diverter Stent Implantation: Risk Factors and Relevance of Standard Observation Time for Early Detection and Management
Source: Clin Neuroradiol. 2022 Sep 6;33(2):343–51. doi: 10.1007/s00062-022-01214-6 (PMC10219871; doi:10.1007/s00062-022-01214-6)
Supplement: Supplementary file 1 — The supplementary material includes a detailed table about the influence of various patient-, aneurysm- and treatment-related variables on the acute intraprocedural thrombus formation. The summary of the univariate models as well as the final multivariable logistic regression model is included. [file 62_2022_1214_MOESM1_ESM.docx]

**SUPPLEMENTAL MATERIAL**

| **Acute thrombosis rate** | | | | | | | | | | | | | | | **P-value (univariate analysis)** |
| --- | --- | --- | --- | --- | --- | --- | --- | --- | --- | --- | --- | --- | --- | --- | --- |
| **Patient-related variables** | | | | | | | | | | | | | | |  |
| **Sex** | Female  8 % | | | | | | | Male  7.7 % | | | | | | | 0.725 |
| **Risk factors** | <50 y  3.7 % | | | | | | | >50 y  7.6 % | | | | | | | 0.226 |
|  | aHT  12.5 % | | | | | | | Normotensive  1.6 % | | | | | | | **0.011** |
|  | DMII  - | | | | | | | Normoglycemic  8.6 % | | | | | | | 0.307 |
|  | Smoker  9.8 % | | | | | | | No history of smoke  2.9 % | | | | | | | 0.088 |
| **SAH** | No  6.8 % | <2 weeks  - | | | | | | >2 weeks  50 % | | | | Acute  - | | | 0.567 |
| **Aneurysm-related variables** | | | | | | | | | | | | | | |  |
| **Location** | ACA / ACOM  18.2 % | MCA 12.5 % | | | | | Intradural ICA  5 % | | | Extradural ICA  14.3 % | | | Vertebrobasilar  4.5 % | | 0.641 |
| **Type** | Bifurcation  12.5 % | | | | | | | Sidewall  6.1 % | | | | | | | 0.252 |
| **Morphology** | Saccular  7.1 % | | | Fusiform  5.3 % | | | | Dissecting  25 % | | | | Blister  - | | | 0.824 |
| **Multiple aneurysms** | Yes  11.9 % | | | | | | | No  5.8 % | | | | | | | 0.278 |
| **No. aneurysms treated simultaneously** | 1  7.6 % | | | | | | | ≥2  11.1 % | | | | | | | 0.615 |
| **Neck width** | < 4 mm  4.2 % | | | | | | | ≥ 4 mm  9.9 % | | | | | | | 0.124 |
| **Maximal diameter** | < 15 mm  6.4 % | | | | | ≥ 15 mm – 24 mm  7.7 % | | | | | ≥ 25 mm  - | | | | 0.754 |
| **Average parent artery diameter** | ≤ 2.5 mm  10.5 % | | | | | | | > 2.5 mm  6.0 % | | | | | | | 0.332 |
| **Side branch coming out of the aneurysm** | Yes  15.2 % | | | | | | | No  5.2 % | | | | | | | **0.041** |
| **Covered side branch of the parent artery** | Yes  7.5 % | | | | | | | No  3.8 % | | | | | | | 0.502 |
| **Treatment-related variables** | | | | | | | | | | | | | | | |
| **Type of FDS** | FRED  5.8 % | | FRED Jr.  21.4 % | | | | | FRED X  10.7 % | | | | PED Vantage  4.8 % | | | 0.647 |
| **No. of simultaneously implanted FDS** | 1  6.2 % | | | | | | | ≥ 2  11.1 % | | | | | | | 0.615 |
| **Pre-interventional antiplatelet therapy** | ASA + Clopidogrel  5.7 % | | | | ASA + Ticagrelor  13.3 % | | | | ASA + Prasugrel  50 % | | | | | Tirofiban*  7.7 % | 0.263 |
| **Clopidogrel-responsiveness** | Yes  5.9 % | | | | | | | No  12.5 % | | | | | | | 0.555 |
| **Technique** | Yes  5.3 % | | | | | | | No  10 % | | | | | | | 0.647 |

**Table 1. Influence of patient-, aneurysm- and treatment-related variables on acute thrombus formation—summary and univariate models**. Abbreviations: year (y); arterial hypertension (aHT); diabetes mellitus type 2 (DM II); subarachnoid hemorrhage (SAH); anterior cerebral artery (ACA); anterior communicating artery (ACOM); middle cerebral artery (MCA); internal carotid artery (ICA); number (no.); flow diverter stent (FDS). * in an acute setting

|  | P Value | Odds Ratio (95% Confidence Interval) |
| --- | --- | --- |
| **aHT** | 0,031 | 9.844 (0.013 - 0.808) |
| **Aneurysm SB** | 0,043 | 3.553 (0.82- 0.963) |

**Table 2.** **Influence of patient- and procedure-related factors on the occurrence of acute intraprocedural thrombosis —final multivariable logistic regression model.** Abbreviations: arterial hypertension (aHT); side branch (SB).
